# Role of Shape in Particle-Lipid Membrane Interactions: From Surfing to Full Engulfment

**DOI:** 10.1021/acsnano.3c11106

**Published:** 2024-03-21

**Authors:** Stijn van der Ham, Jaime Agudo-Canalejo, Hanumantha Rao Vutukuri

**Affiliations:** † Active Soft Matter and Bio-inspired Materials Lab, Faculty of Science and Technology, MESA+ Institute, University of Twente, 7500 AE Enschede, The Netherlands; ‡ Department of Living Matter Physics, Max Planck Institute for Dynamics and Self-Organization, Göttingen, D-37077, Germany; ¶ Department of Physics and Astronomy, University College London, London WC1E 6BT, United Kingdom

**Keywords:** Vesicles, Lipid membranes, Anisotropic particles, Wrapping, Passive engulfment, Cellular particle uptake

## Abstract

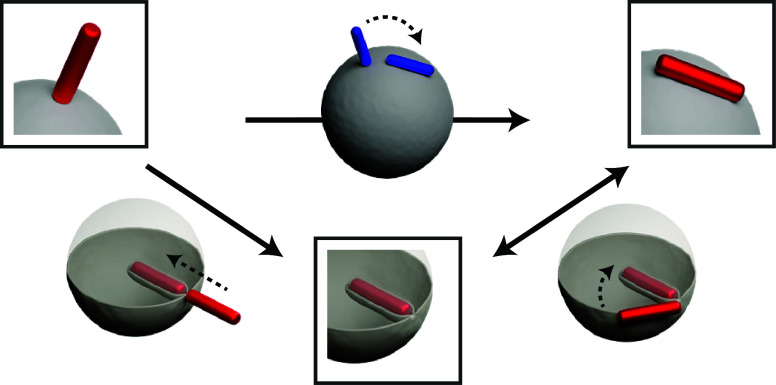

Understanding and manipulating the interactions between foreign bodies and cell membranes during endo- and phagocytosis is of paramount importance, not only for the fate of living cells but also for numerous biomedical applications. This study aims to elucidate the role of variables such as anisotropic particle shape, curvature, orientation, membrane tension, and adhesive strength in this essential process using a minimal experimental biomimetic system comprising giant unilamellar vesicles and rod-like particles with different curvatures and aspect ratios. We find that the particle wrapping process is dictated by the balance between the elastic free energy penalty and adhesion free energy gain, leading to two distinct engulfment pathways, tip-first and side-first, emphasizing the significance of the particle orientation in determining the pathway. Moreover, our experimental results are consistent with theoretical predictions in a state diagram, showcasing how to control the wrapping pathway from surfing to partial to complete wrapping by the interplay between membrane tension and adhesive strength. At moderate particle concentrations, we observed the formation of rod clusters, which exhibited cooperative and sequential wrapping. Our study contributes to a comprehensive understanding of the mechanistic intricacies of endocytosis by highlighting how the interplay between the anisotropic particle shape, curvature, orientation, membrane tension, and adhesive strength can influence the engulfment pathway.

## Introduction

Endocytosis is a fundamental cellular process that mediates the uptake of nutrients, pathogens, and therapeutic agents.^1^ Understanding and controlling the interaction between foreign bodies and cell membranes in endocytosis are critical for numerous biomedical applications, including targeted drug delivery,^2^ intracellular imaging,^3^ and nanotoxicity studies.^4^ Biomimetic model systems such as giant unilamellar vesicles (GUVs) offer a pathway to a comprehensive understanding of this process.^5−7^ GUVs are extensively used to investigate passive engulfment, a process where particle wrapping is driven by generic physical interactions like particle–membrane adhesion.^8−15^ Particle wrapping is dictated by the balance between the adhesion free energy gain from particle–membrane overlap and the elastic free energy cost incurred by membrane deformation.^12,16,17^

Various studies to date have investigated the role of different parameters influencing the wrapping process, such as particle size,^17,18^ membrane asymmetry,^18,19^ membrane tension and fluctuations,^16,20^ local membrane curvature,^18,21−23^ particle surface properties,^9^ and notably, particle shape.^13,24−33^ Particle shape has attracted considerable attention given its impact on the bending free energy cost and potential to modify the engulfment pathway. For example, several theory and simulation studies predict that anisotropic particles, such as ellipsoids and rods, experience a spontaneous rotation during engulfment, a phenomenon that reduces the bending free energy cost.^26,28,29,33^ On the other hand, only a limited number of experimental studies have investigated the role of shape anisotropy in the wrapping process, for example, using DNA origami rods,^31^ microgel particles,^32^ or dumbbell particles^13^ with GUVs, gold nanoparticles with HeLa cells,^24^ and carbon nanotubes with mammalian cells.^34^ However, these studies are limited by using either nontunable^32^ and strong binding interactions (e.g., NeutrAvidin-biotin binding^13^) or relatively small particles,^24,31,34^ which makes tuning and following their membrane interactions at the single-particle level challenging. A detailed understanding of how rod-like particles interact with membranes during endocytosis not only is of fundamental interest but also has important toxicological implications. This is particularly evident in studies on the size-dependent phagocytosis of asbestos rods by macrophages, which is a critical factor in asbestos-related toxicology research.^35,36^ Additionally, the role of membrane tension, crucial in processes like inhibiting parasite infection when elevated,^37^ is often neglected in experimental minimal model systems.

In this study, we comprehensively investigate the role of the anisotropic particle shape, curvature, orientation, membrane tension, and adhesive strength on the wrapping pathway at a single-particle level. We employ both straight and curved rods with flat and round tips, along with a nonadsorbing polymer that enables tunable, nonspecific, adhesive interactions via depletion forces.^8,11,38^ Our model system offers excellent control over the particle’s wrapping pathway by the lipid membrane, spanning a range of interactions from a surfing state, where the particle adheres to the membrane without inducing deformation, to partially and fully wrapped states.

We find that the interplay between the elastic free energy penalty and the free energy gain from depletion attractions controls the wrapping of rod-like particles. Interestingly, we observe two distinct engulfment pathways: tip-first, where the rod’s long axis remains perpendicular to the vesicle membrane, and side-first, where the rod’s long axis starts parallel to the membrane and rotates to a perpendicular orientation as the degree of wrapping increases. The initial orientation of the rod relative to the membrane plays a crucial role in determining which pathway the rod follows.

The paper is organized as follows: we begin by exploring rod-membrane interactions in the high-tension vesicle regime. We then focus on the engulfment pathways of the rods, investigating the effects of rod curvature, aspect ratio, adhesion strength, and membrane tension on these pathways. Following this, we examine the sequential engulfment of clusters involving multiple rods. Finally, corroborating our experimental observations with numerical calculations, we constructed a state diagram that encapsulates these findings.

## Results and Discussion

Our experimental model system consisted of three main components: giant unilamellar vesicles (GUVs), rod-shaped particles with different curvatures and aspect ratios, and a nonadsorbing polymer. GUVs of 1,2-dioleoyl-*sn*-glycero-3-phosphocholine (DOPC) lipids were produced with the droplet transfer method,^7,39^ resulting in vesicles with varying size and membrane tensions.

**Figure 1:**
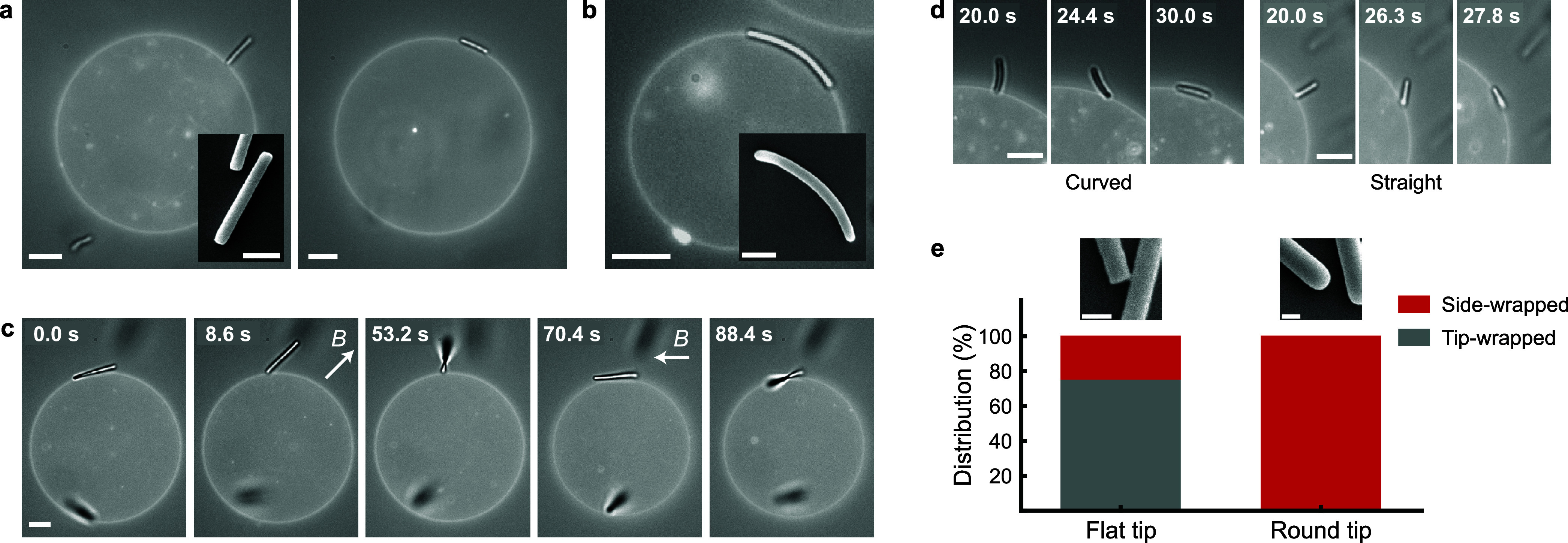
Partially wrapped state. Combined fluorescence and bright-field microscopy images illustrating the partially wrapped state of rods: (a) Flat-tipped rods in the tip-wrapped (left panel) and side-wrapped (right panel) state. (b) Round-tipped rod with vesicle-matching curvature in the side-wrapped state. The insets are scanning electron microscope (SEM) images of a straight and curved rod, respectively. (c) Time-lapse images depicting a magnetically responsive flat-tipped rod transitioning from the side-wrapped to the tip-wrapped state and vice versa. The magnetic field is denoted by the letter B, and the arrow indicates the direction of the applied magnetic field along which the rod aligns. Between transitions, the magnet was removed to demonstrate the stability of the rod in its new orientation. (d) Time-lapse images showing the spontaneous transition of a round-tipped curved and straight rod from the tip-wrapped to the side-wrapped state. (e) The distribution of rods over the tip-wrapped and side-wrapped state as a function of tip shape, 5 min after their initial adhesion to the vesicle. The scale bars in the microscopy images and insets represent 10 and 1 μm (a–d), respectively. Scale bars in the SEM image insets (e) represent 0.4 μm.

We employed two fabrication methods to produce the rods from SU-8 photoresist^40,41^ (see the Methods section and the Supporting Information (SI), Section S1 and Figure S1). The first method produces straight rods with flat tips (Figure 1a), characterized by an aspect ratio (defined as *h*/*a*, where *h* is the length and *a* is the radius) ranging from 5 to 60. In contrast, the second method generates curved rods with round tips (Figure 1b). Importantly, the second method can also produce “straight” rods with round tips, as it allows for the fabrication of a range of curvatures (radius of curvature ranges from approximately 2 to 50 μm^–1^ with opening angles from 4 to 194°). Therefore, throughout this paper, we employ “straight” and “curved” to refer to the rods’ long axis curvature, and we explicitly specify the tip shape when it is necessary for clarity.

In the absence of a nonadsorbing polymer, the rods did not show any specific interactions with the lipid membrane (SI, Figure S3). Therefore, to induce an adhesive interaction between the rod and the membrane, we used polyacrylamide (PAM, with a molecular weight of 700,000–1,000,000 g/mol) as a nonadsorbing polymer to serve as a depletant. In the dilute limit, the depletion free energy is given by *E*_ad_ = *n*Δ*Vk*_B_*T*,^38^ where *n* is the number density of depletant, and Δ*V* is the change in the depletant’s excluded volume. An effective rod-membrane attraction arises from the reduction of the excluded volume due to an increase in the overlap volume as the rod and membrane come into contact. We estimate Δ*V* as the overlap volume, *V*_ov_, between the excluded volume regions of the rod and the vesicle membrane. The overlap volume *V*_ov_ can be calculated using *V*_ov_ = 2*R*_G_*A*_co_, where *R*_G_ is the radius of gyration of the depletant, and *A*_co_ is the contact area between the rod and the membrane (for more details see the SI, Section S2 and Figure S2). The adhesive strength, *W*, is given by *W* = *E*_ad_/*A*_co_ = 2*R*_G_*nk*_B_*T* and can be tuned by varying the PAM concentration (0.25–0.75 wt %). We studied the resulting dynamic interactions between the rods and the membrane using an inverted fluorescence and a confocal scanning laser microscope.

### Partially Wrapped State

For high-tension vesicles (≥*O* (10^–6^ N/m)), we observed rods in a partially wrapped state across a range of adhesive strengths (0.25–0.75 wt % PAM). Owing to the high free energy penalty for deforming the membrane, these rods only induced a small membrane deformation relative to their unbound state. Consequently, only a small fraction of the rod’s surface area was wrapped, referred to as a shallow-wrapped state (see Figure S2b for a schematic depiction). Interestingly, we observed two distinct configurations in which the rods were partially wrapped by the vesicle membrane, as illustrated in Figure 1a (Movie S1). In the first configuration, the rod adheres to the membrane with its tip (i.e., short axis) and its long axis oriented perpendicular to the vesicle membrane. We refer to this as the tip-wrapped state (Figure 1a). Conversely, in the second configuration, the rod adheres to the membrane with its side and its long axis parallel to the membrane. We term this as the side-wrapped state (Figure 1a,b).

The variation in rod orientation results in a larger membrane-rod overlap area in the side-wrapped state, inducing a stronger adhesive interaction via depletion attractions compared to the tip-wrapped state. As a result, rods in the side-wrapped state bind more strongly to the vesicle membrane than those in the tip-wrapped state. At a low depletion attraction strength (<0.5 wt % PAM), our observations suggest that rods in the tip-wrapped state are prone to detaching after several seconds due to thermal fluctuations. However, the rods in the side-wrapped state maintain a strong attachment after initial contact. Nonetheless, at higher polymer concentrations (≥0.5 wt %), even the rods in the tip-wrapped state maintain a strong attachment to the vesicle membrane.

We observed spontaneous transitions of rods between the two partially wrapped states occurring only in one direction, from tip-wrapped to side-wrapped. This likely stems from the significantly higher adhesion strength present in the side-wrapped state (the overlap volume in side-wrapped state is 10 times higher than in the tip-wrapped state for *h* = 5 μm and *a* = 0.2 μm, see the SI, Section S8). Interestingly, the occurrence of this transition and the stability of the tip-wrapped state strongly depended on the shape of the rod’s tip. We observed that the majority of rods with round tips spontaneously transitioned from the tip-wrapped to the side-wrapped state due to thermal fluctuations, as depicted in Figure 1d and Movie S2. Conversely, rods with flat tips exhibited greater stability in the tip-wrapped state and infrequently transitioned to a side-wrapped configuration.

To further probe stability, we used magnetically responsive rods, which allowed us to manipulate their position and orientation using an external magnetic field. Using this magnetic field, we positioned the rods in the tip-wrapped state by adhering one of their tips to the vesicle (0.5 wt % PAM). After the magnet was removed, we measured the time until a transition to the side-wrapped state occurred. The distribution of rods in the tip-wrapped and side-wrapped states after 5 min is depicted in Figure 1e for rods with round and flat tips, respectively. It should be noted that we monitored the rods’ orientation for 10 min. However, no further changes in the distribution over partially wrapped states were observed after the initial 5 min (see the SI, Figure S4).

The distribution reveals that rods with round tips transition to the side-wrapped state at a rate significantly higher than that of those with flat tips. To decouple any effects of lengthwise curvature, we incorporated both straight and curved rods with round tips in our analysis, as illustrated in Figure 1d and the SI, Figure S5. The findings indicate no significant dependence on the rods’ long axis curvature, suggesting that the tip shape is the decisive factor in determining the stability of the tip-wrapped state.

To delve deeper into how particle shape affects the stability of the partially wrapped state, we again employed magnetically responsive rods, this time manipulating their orientations in order to induce a transition. We found that, with the application of an external magnetic field (∼2–10 mT), flat-tipped rods were able to reversibly transition between the side-wrapped and tip-wrapped states, as shown in Figure 1c (Movie S3). After each transition, the magnetic field was removed to evaluate the stability of the rod in its new configuration. Notably, a rod could transition from the tip-wrapped state to the side-wrapped state if it was sufficiently tilted relative to the vesicle membrane. However, transitions from the side-wrapped state to the tip-wrapped state were seldom observed, owing to the high adhesion strength in the side-wrapped state and necessitated a significant magnetic field strength.

These experiments indicate the presence of a free energy barrier between a stable side-wrapped state and a metastable tip-wrapped state, with its magnitude dependent on both the transition direction and the shape of the rod’s tip. We observe no spontaneous transitions from side-wrapped to tip-wrapped states, implying a substantial free energy barrier. However, transitions from tip- to side-wrapped states were frequent for rods with round tips, suggesting no free energy barrier or one that is small compared to the thermal energy. In contrast, flat-tipped rods require rotational manipulation via a magnetic force to undergo the same transition, signifying a higher, yet surmountable, free energy barrier.

To corroborate our findings, we performed a quantitative analysis to assess the overlap volumes and thus the adhesive strength between the rods and vesicle membranes (SI, Section S8), which consistently supports our qualitative observations. Importantly, this analysis elucidates how the rod’s tip shape affects free energy barrier heights during transitions from tip- to side-wrapped states. In the transition state between the tip-wrapped and side-wrapped states, a diagonally oriented rod with a flat tip shape leads to a reduced excluded volume overlap, thus creating a free energy barrier. In contrast, a rod with a round tip maintains this overlap, resulting in no free energy barrier. The presence or absence of this barrier significantly influences the stability of the tip-wrapped state, thereby explaining the more frequent occurrence of this state in flat-tipped rods.

### Fully Wrapped State

**Figure 2:**
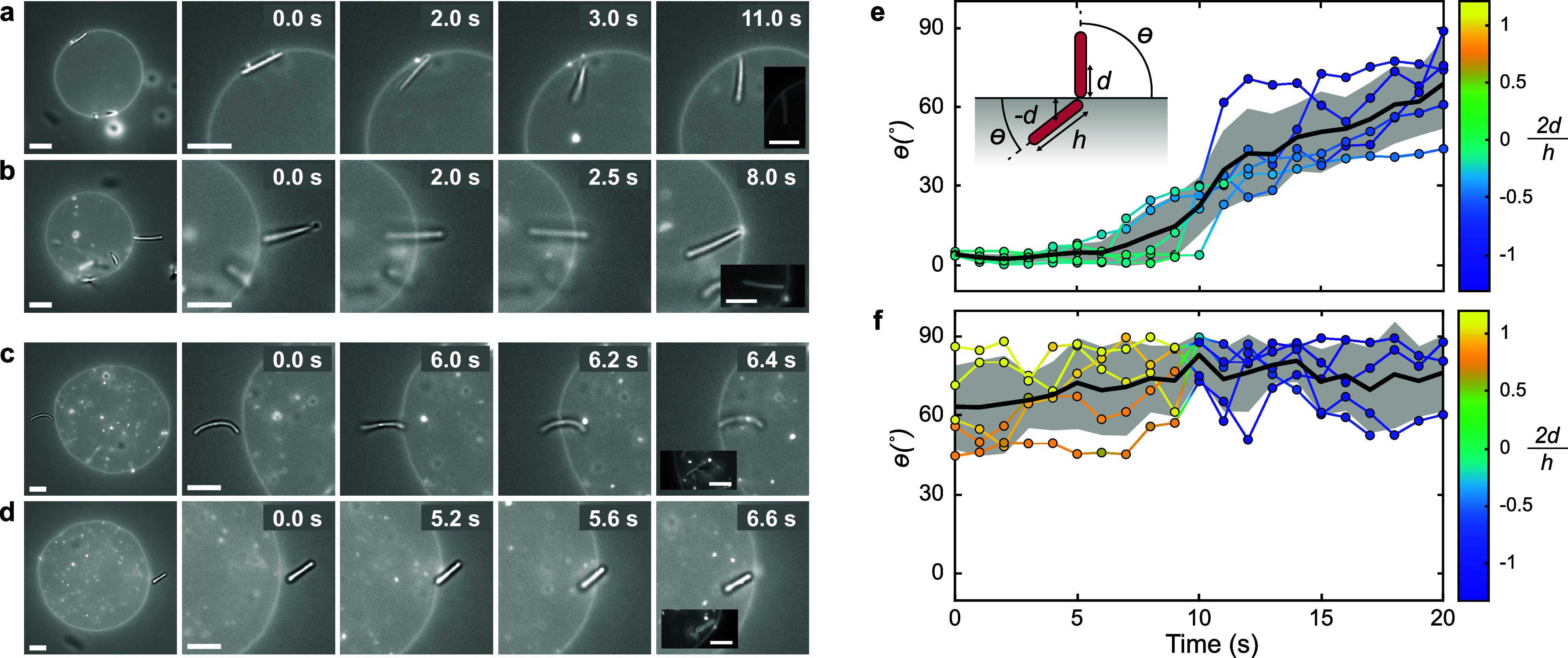
Wrapping pathways. Time-lapse of combined fluorescence and bright-field microscopy images depicting the engulfment pathways. Insets present the fluorescence image exclusively, showcasing the membrane morphology. (a) Side-first engulfment of a round-tipped rod by a GUV: initially in the side-wrapped state, the rod transitions to the fully wrapped state. As the degree of wrapping increases, the rod’s long axis rotates from being parallel to becoming perpendicular to the GUV membrane. (b) Tip-first engulfment of a flat-tipped rod by a GUV: initially adhered in the tip-wrapped state, the rod transitions to the fully wrapped state. The rod’s long axis remains perpendicular to the GUV membrane during this transition. (c, d) Tip-first engulfment involving a curved rod with round tips (c) and a straight rod with flat tips (d) by a vesicle under low tension: upon making contact with the membrane, the rod is instantly engulfed, transitioning from the free state to the fully wrapped state. All scale bars represent 5 μm. (e, f) The angle and distance between the rod and the membrane as a function of time during side-first engulfment (e) and tip-first engulfment (f). For each pathway, five independent measurements are plotted. The black line with the gray shaded area represents the average and standard deviation of these measurements, respectively. The inset in (e) provides a schematic depiction of the definitions of the angle θ and the distance *d*.

We increased the PAM concentration to further probe the role of adhesive strength in the wrapping process. For vesicles at intermediate tension (*O* (10^–7^ N/m)), this increase led to a transition of rods from a partially wrapped to a fully wrapped state (see SI, Figure S2c) at higher adhesive strengths (0.66–0.75 wt % PAM). This transition was discontinuous and transpired rapidly within a few seconds or less. Interestingly, we observed that this process occurred along two distinct pathways:

(i) The rod is initially attached in the side-wrapped state and hinges into the GUV (see Figure 2a and Movie S4). As it transitions from side-wrapped to fully wrapped, the rod reorients itself from parallel to perpendicular relative to the membrane. This reorientation occurs due to the considerable bending free energy cost associated with enveloping both highly curved tips. Instead, the rod undergoes a rotational motion during the wrapping process such that only one tip is fully wrapped, while the other remains positioned near the membrane. These findings align with simulation and numerical predictions,^26,28−30,33^ which typically show the engulfment of ellipsoidal and spherocylindrical particles starting with a lateral attachment to the membrane and followed by a transition to a perpendicular orientation as wrapping advances. A similar rotational behavior has been observed in experiments involving the wrapping of dumbbell-shaped particles by Azadbakht et al.^13^ However, it is crucial to distinguish that in their study rotation is prompted by the inhomogeneous ligand coating distribution on the particle. In contrast, our study demonstrates that the rod’s rotation is solely driven by variations in particle curvature, emphasizing the significance of curvature in this process.
(ii) Alternatively, the rod begins in the tip-wrapped state, docking to the membrane with its tip, and is then engulfed into the GUV without undergoing reorientation (Figure 2b and Movie S4). This reflects the findings of Dasgupta et al.,^28^ who predicted that short rods with flat tips enter tip-first via a rocket-like pathway, whereas rods with higher aspect ratios or more rounded tips exhibit side-first entry, undergoing a rotation throughout the wrapping process. However, our findings extend beyond this model, as we observe also high aspect ratio rods (*h*/*a* = 10–60) with flat tips stably adhering in the tip-wrapped state and undergoing tip-first entry (Figures 1a, 2b and SI, Figure S7). This aligns with the work of Shi et al.,^34^ which suggests a similar entry mode for high aspect ratio carbon nanotubes in mammalian cells. We postulate that the increased stability of the tip-wrapped state is due to a free energy barrier between the two partial wrapping states, generating a local minimum and enhancing the state’s stability.

To quantify the two engulfment pathways, we measured both the angle (θ), and the shortest distance (*d*), between the rod’s center of mass and the vesicle membrane during engulfment. It should be noted that θ is considered a positive value regardless of the rod’s orientation relative to the membrane, while *d* is positive when the rod’s center of mass is outside the vesicle and negative when inside. For more details on the measurement techniques for θ and *d*, see SI, Section S3.

A total of five transitions for each pathway were measured, as shown in Figure 2e,f. The two pathways display distinctive angle progressions: side-first engulfment exhibits a rotation from 0° to approximately 45–90°, while tip-first engulfment maintains rods at an angle between 45 and 90° throughout the process. Our analysis indicates that both pathways are accessible to rods of various lengths and tip shapes. Side-first engulfment is observed for both curved and straight rods with radii of curvature from 9 to 31 μm and aspect ratios (*h*/*a*) from 12 to 45. Conversely, tip-first engulfment is predominantly observed for straight rods, with aspect ratios from 10 to 31. Despite the intrinsic error in measuring angles and distances due to the 2D projection of a 3D system, the angle and distance progressions are consistent, and independent of variations in rod aspect ratio, curvature, and tip shape.

The vesicle membrane morphology when rods are in the fully wrapped state can be inferred from the fluorescence image, as depicted in the insets of Figure 2a,b. The combined bright-field and fluorescent image distinctly shows both the lipid membrane and the rod, whereas the fluorescent mode exclusively highlights the lipid membrane, owing to the nonfluorescent nature of the rods. This visual evidence affirms that the rod is indeed in a fully wrapped state. Although limitations in spatial resolution make it challenging to definitively identify a membrane neck connecting the rod to the membrane, we observe that the rods remain close to the membrane and are typically oriented at an angle of approximately 45–90° to the membrane (Figure 2e,f). This suggests the presence of a narrow neck connecting them at their tips, inferred from the minimal membrane deformation observed near the rod tip.^42^

It is worth noting that, in addition to a “shallow” partially wrapped state (Figure 1) and a fully wrapped state (Figure 2), also a “deep” partially wrapped state is predicted for rod-shaped particles^28,33^ (see SI, Section S2 and Figure S2). However, we observe that rods transition directly from the shallow to the fully wrapped state, despite only a minor gain in excluded volume overlap between the deep and fully wrapped states. We hypothesize that the formation of a catenoidal membrane neck in the fully wrapped state minimizes the vesicle membrane’s elastic energy, thus contributing to the completion of the rod wrapping, similar to the engulfment of spherical particles as predicted by Deserno.^16^

### Cooperative Wrapping

**Figure 3:**
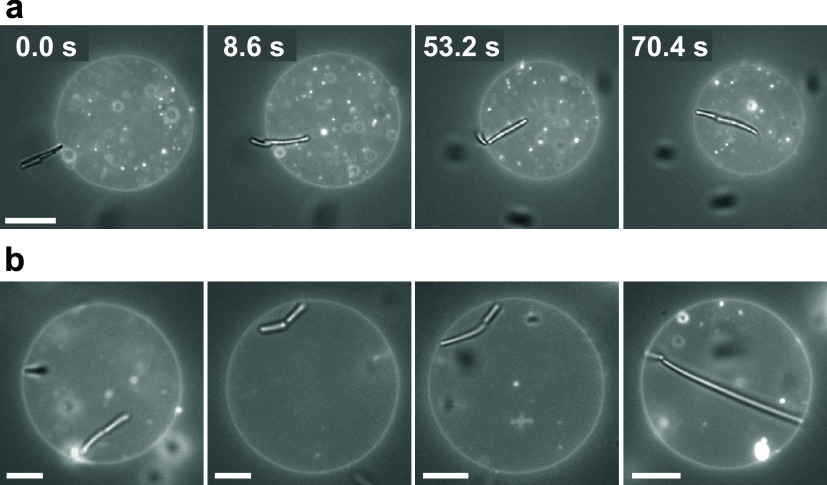
Cooperative wrapping of rod clusters. (a) Time-lapse of overlaid fluorescence and bright-field microscopy images revealing the stepwise cooperative wrapping of a two-rod cluster (round-tipped). (b) Composite (bright-field + fluorescence) microscopy images of rods oriented tip-to-tip in the fully wrapped state. The scale bars represent 10 μm.

At particle concentrations of (∼1.0 wt %), we observed rod clusters forming due to nonspecific depletion interactions, which promote a side-by-side arrangement resulting from the increased overlap volume between individual rods. At PAM concentrations of 0.5 wt % or higher, these rod clusters exhibited cooperative and sequential wrapping. For instance, a cluster featuring a smaller rod attached to the end of a longer one is depicted in Figure 3a. Upon contacting a low-tension vesicle, the longer rod was rapidly engulfed, while the shorter rod slid off and remained on the vesicle surface in a side-wrapped state. After several minutes, the shorter rod transitioned from the side-wrapped to the fully wrapped state, forming a tube-like structure that encapsulated both rods, as shown in Movie S5.

Cooperative wrapping of spherical particles has been previously predicted by Bahrami et al.^43^ to be energetically favorable compared to individual particle wrapping. In related work, Raatz et al.^44,45^ additionally observed that an increased interaction range promotes cooperative wrapping. Furthermore, the effect was found to be more pronounced for prolate particles, as their strongly curved tips do not need to be wrapped within a tubular structure, as demonstrated by Xiong et al.^46^ Therefore, the finite range (*R*_G_ ≈ 50 nm) of the depletion interaction and the prolate shape of the rods in our experiments appear to contribute to the cooperative wrapping of rods in membrane tubes. In the final wrapped configuration, the rods are oriented with their highly curved tips pointing toward each other. This configuration, with both rods wrapped within a single membrane tube, is frequently observed, irrespective of the rods’ relative aspect ratios, as illustrated in Figure 3b.

### Vesicle Membrane Tension

To examine the effect of membrane tension on the wrapping process, we varied the tension of the GUVs. In the low tension regime (*O* (10^–8^ N/m), as measured by shape fluctuation analysis via flickering spectroscopy, see Methods), we observed that full engulfment bypasses the intermediate partially wrapped state. Instead, at intermediate adhesive strengths and higher (≥0.5 wt % PAM), rods are spontaneously engulfed by the vesicle upon contact with the membrane. This is demonstrated in Figure 2c,d and illustrated in Movie S6. Notably, this engulfment behavior, which occurs without the rod needing to reorient, is consistent across both curved and straight rods, regardless of whether an external magnetic field is applied to position the rod near the vesicle membrane.

GUVs with low membrane tension facilitate rod engulfment, while a higher tension opposes it. To explore this further, we increased the membrane tension via a hypotonic shock (detailed in the SI, Section S4) and observed rods that were initially in a fully wrapped state at 0.5 wt % PAM. The resulting osmotic pressure difference between the interior and exterior of the vesicle leads to an increase in membrane tension, causing the particle to rapidly transition from fully wrapped to partially wrapped. We note that these results are consistent with the simulation predictions by Yu et al.^47^ With increased tension, rods previously in a fully wrapped state transitioned back to a side-wrapped state, requiring a rotational movement (as shown in the SI, Figure S8 and Movie S7). We did not observe any transitions to a tip-wrapped state nor complete detachment of the rods from the vesicle, which aligns with expectations given the constant PAM concentration during vesicle inflation. Following the expulsion, the rod rapidly reattached to the vesicle, suggesting that it remained adhered to the membrane.

### State Diagram

**Figure 4:**
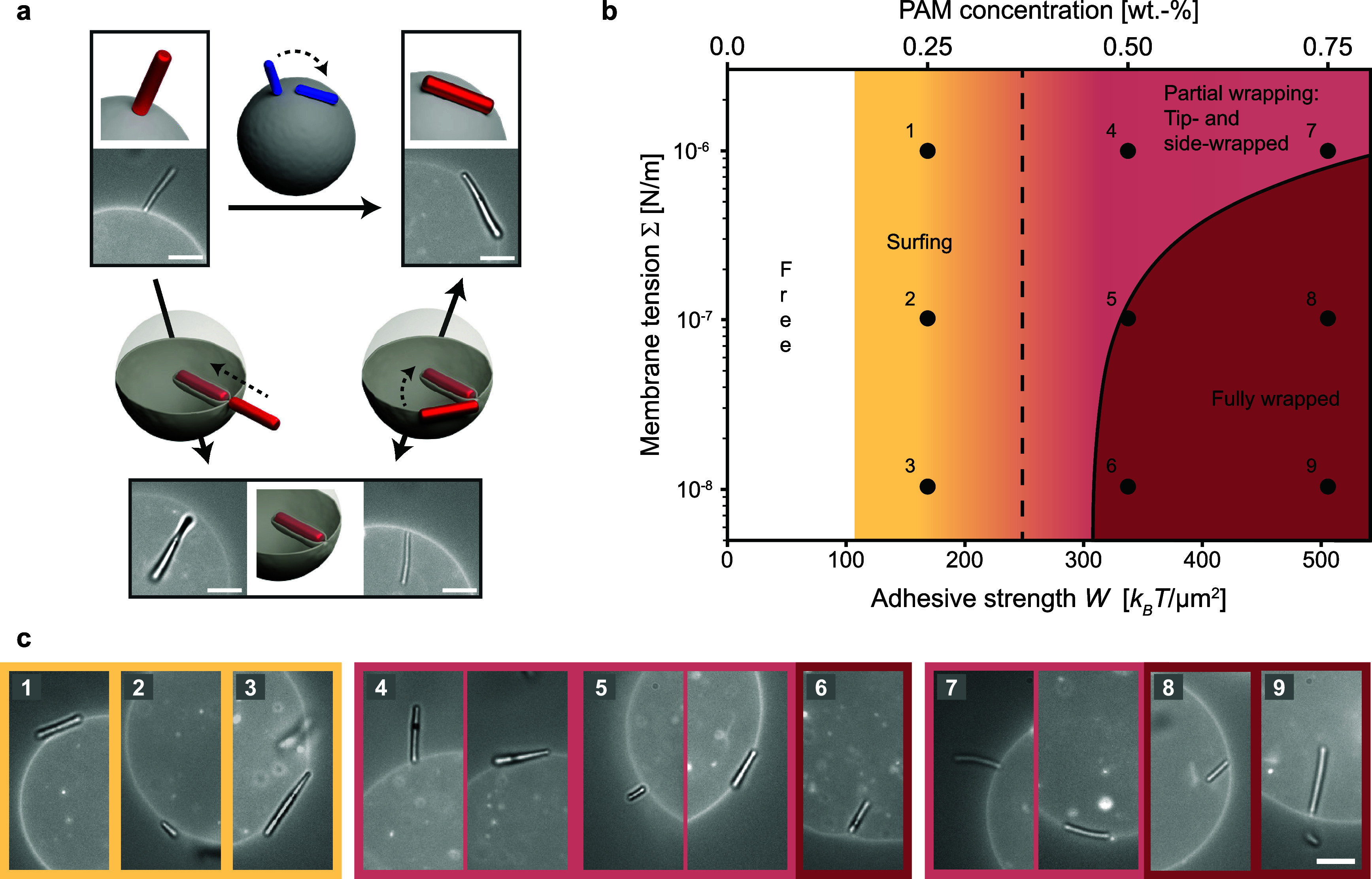
State diagram. (a) Schematic illustration of transitions between the tip-, side-, and fully wrapped states, with arrows indicating the possible directions of transition pathways. The transition from the tip- to the side-wrapped state was predominantly observed for rods with round tips (depicted in blue), whereas all other transitions were seen for both flat- and round-tipped rods (depicted in red). (b) State diagram depicting the experimentally observed and theoretically predicted wrapping states as a function of adhesion strength *W* and membrane tension Σ. The vertical dashed line indicates the theoretical transition from the free to the side-wrapped state, as defined by *W* > κ/(2*a*^2^), where *a* = 0.2 μm is the rod radius. The curved solid line denotes the condition *W* > *E*_be_/*A* + Σ, where *E*_be_ is the total bending free energy and *A* represents the area of the rod shape, respectively (SI, Section S7). (c) Composite (bright-field + fluorescence) microscopy images showcasing the experimentally observed states. Note that these experimental observations qualitatively correspond to the state diagram and are included for illustrative purposes. Their placement within the diagram does not indicate the exact tension of the GUVs; instead, we estimate the tension to be Σ = *O* (10^–6^ N/m), *O* (10^–7^ N/m), or *O* (10^–8^ N/m), based on the shape fluctuations of the vesicles (SI, Figure S10). For locations 4, 5, and 7, images illustrate both the tip-wrapped state (left) and the side-wrapped state (right). The scale bars represent 5 μm.

Figure 4 presents a summary of the experimental results for different adhesive strengths and membrane tensions. Figure 4a illustrates the observed pathways among the three states, while Figure 4b,c displays a state diagram integrating experimental results with numerical calculations.

Following ref (29), the critical adhesion strength for the transition from the free to the partially wrapped state is given by *W* > 2*κM*_pa_^2^, where *W* is the adhesive strength, κ ≈ 20 *k*_B_*T* is the bending rigidity,^7^*M*_pa_ is the local mean curvature of the particle at the point of membrane contact, and we have neglected the local curvature of the membrane, which is much smaller than that of the particle. Since κ is a material constant, the critical adhesion solely depends on the local mean curvature *M*_pa_ of the particle. Thus, as we move along the horizontal axis in Figure 4b from low to high adhesive strength, we expect flat-tipped rods to initially adhere in the tip-wrapped orientation (with *M*_pa_ ≈ 0, implying *W* ≳ 0), followed by round- and flat-tipped rods in the side-wrapped orientation (with *M*_pa_ = 1/(2*a*), where *a* represents the rod radius, implying *W* > κ/(2*a*^2^)^48^) (SI, Section S7).

Surprisingly, our experimental observations contradict these predictions, showing that side-wrapping occurs first with an increase in adhesion strength for flat-tipped rods (see Figure 4c). This discrepancy can be attributed to the interaction range between the rods and the membrane.^44,49^ The theoretical prediction above assumes a zero interaction range between the rod and the membrane, while in our experiments, this range is on the order of the depletant size (*R*_G_ ≈ 50 nm). This extended interaction range enables rods to adhere to the membrane without necessitating membrane deformation. Consequently, we observe an intermediate regime between the free and partially wrapped states, where the rod adheres to the vesicle membrane in a side-oriented state without deforming the membrane. We will refer to rods in this state as surfing (SI, Section S8).

From the partially wrapped state, increasing the adhesion strength or decreasing membrane tension leads to a transition to the fully wrapped state. This transition is discontinuous and estimated to occur in the region where *W* > *E*_be_/*A* + Σ, where *E*_be_ is the bending free energy of the membrane wrapped around the rod, *A* is the surface area of the rod, and Σ is the membrane tension (SI, Section S7). This transition is essentially an energetic condition and does not account for the possible existence of a free energy barrier between the partially and fully wrapped states, which may be quite large and only vanishes at even larger adhesion strengths, as has been shown for ellipsoidal particles in ref (29).

Nevertheless, the experimental observations qualitatively align well with these predictions. Notably, within the explored range of depletion strengths, high-tension vesicles (≥*O* (10^–6^ N/m)) do not attain a fully wrapped state, as the membrane tension’s free energy cost supersedes the gain in adhesion. Conversely, low-tension vesicles (*O* (10^–8^ N/m)) achieve a fully wrapped state at intermediate adhesion strengths and above (≥0.5 wt % PAM). Note that vesicles with low tension possess an excess membrane area compared to a sphere with an identical volume, whereas those with high tension do not. We believe that both the volume and surface area of the vesicle are constant during rod engulfment. This can be attributed to the fact that the excess membrane area ensures that the total membrane area is constant, while imposed isomolar conditions maintain a constant volume. Finally, for vesicles with intermediate tension, achieving a fully wrapped state necessitates higher adhesion strengths (above 0.5 wt %).

## Conclusion

In conclusion, our research elucidates the critical role of the anisotropic particle shape, orientation, curvature, membrane tension, and adhesive strength in the engulfment process. We demonstrate how to control the particle engulfment, spanning a range of interactions from surfing to partially to fully wrapped states, orchestrated by the interplay between the nonspecific adhesive strength and the elastic free energy penalty. In the partially wrapped state, we find that the tip shape determines the stability of the tip-wrapped state, highlighting the importance of the curvature of the tip in the wrapping process. Our quantitative analysis reveals that rods with flat tips exhibit stable adhesion in this state, while those with rounded tips tend to transition to the side-wrapped state.

Our study identifies two distinct engulfment pathways by which rod-shaped particles can achieve a fully wrapped state: rods that initially adhere with their tips follow a tip-first path, while those adhering laterally take a side-first path, involving a rotation of the rod as the degree of wrapping advances. Notably, our findings indicate that the angle progression of rods during engulfment remains consistent over a range of rod aspect ratios and curvatures, indicating that the pathways are determined by particle shape anisotropy rather than the rod’s long-axis curvature and aspect ratio. Furthermore, under very low membrane tension conditions, rods directly pursue a tip-first pathway to a completely wrapped state, irrespective of their aspect ratio and curvature, emphasizing the crucial influence of the membrane tension in the engulfment process. Next, we find that when multiple rods are in a fully wrapped state, they reach a tube-like structure, pointing their highly curved tips toward each other to minimize the bending free energy. Our experimental results are consistent with theoretical predictions in a state diagram, illustrating how to control the wrapping pathway from surfing to partial to complete wrapping by modulating membrane tension and adhesive strength.

Overall, our research contributes to a broader understanding of anisotropic particle engulfment pathways and their significance in endocytosis and phagocytosis,^50^ extending implications for advanced biomedical applications such as targeted drug delivery, intracellular imaging, and the critical area of nanotoxicity studies.^35,36^ While our experiments employed a simplified biomimetic system, the principles we have established could be applied to more complex environments in future research, potentially incorporating diverse lipid compositions, membrane proteins, and intricate particle geometries to further dissect the complexities of particle engulfment.

## Methods/Experimental Section

### Materials

All chemicals, unless otherwise specified, were used as received. 1,2-Dioleoyl-*sn*-glycero-3-phosphocholine (DOPC) and fluorescent 1,2-dioleoyl-*sn*-glycero-3-phosphoethanolamine-*N*-(lissamine rhodamine B sulfonyl) (ammonium salt) (Liss Rhod PE) in chloroform were obtained from Avanti Polar Lipids (Alabaster, AL). Chloroform (≥99.5%), paraffin oil, glucose, sucrose, glycerol (≥99.0%), iron(II, III) oxide nanopowder 50–100 nm, and PEG-PPG-PEG Pluronic F-108 were obtained from Sigma-Aldrich. SU-8 50 photoresist was procured from Microchem. A solution of 10 wt % polyacrylamide (PAM) (MW 700,000–1,000,000) in water was obtained from Polysciences Inc.

### Vesicle Preparation Protocol

Lipid-oil solution (LOS) was prepared following an adapted protocol by Vutukuri et al.^7^ 1,2-Dioleoyl-*sn*-glycero-3-phosphocholine (DOPC) and fluorescent 1,2-dioleoyl-*sn*-glycero-3-phosphoethanolamine-*N*-(lissamine rhodamine B sulfonyl) (ammonium salt) (Liss Rhod PE) were diluted in chloroform to final concentrations of 12 mg/mL (15 mM) and 0.2 mg/mL (0.15 mM), respectively, and stored at −20 °C until use. To prepare LOS, 0.31 g of DOPC and 0.12 g of Liss Rhod PE stock solution were added to a clean glass vial (Sample Storage Assembled Screw Vial Kits, Thermo Scientific). The chloroform was then evaporated under a gentle N_2_ airflow while rotating the vial to create an even layer of dried lipids on the bottom. The vial was placed in a desiccator for 2 h to remove any remaining traces of chloroform. Subsequently, 2.2 g of paraffin oil was added to the vial, followed by sonication for 1 h while heating the bath to 60 °C to enhance lipid solubilization. The LOS was then kept in a 60 °C oven overnight to ensure complete dissolution of the lipids and later stored at the same temperature.

Vesicles were prepared using the droplet transfer method.^7,39,51^ The inner and outer solutions were composed of 100 mM sucrose and 100 mM glucose, respectively. In a 2 mL Eppendorf tube, 200 μL of LOS was layered on top of 500 μ L of the outer solution. In a separate 2 mL Eppendorf tube, 600 μL of LOS was mixed with 100 μL of the inner solution for 2–3 min using a 1 mL pipet to create an emulsion. Next, 120 μL of the emulsion, taken from the top of the second tube, was added to the water–oil interface in the first tube. The mixture was then immediately centrifuged (Centrifuge 5425, Eppendorf) at 200*g* for 2 min, thus forming vesicles. Using a pipet, the top oil layer was carefully removed, leaving the vesicle solution at the bottom of the tube. The tube was left undisturbed for 30–60 min to allow the vesicles to accumulate before transferring them to the imaging chamber.

### Rod Preparation Protocol

Curved and straight microrods were fabricated following an adapted protocol for the formation of SU-8 rods,^40,41,52^ based on earlier work from Alargova et al.^53^ In a typical synthesis, 110 mL of glycerol was added to a 250 mL glass beaker. A mixer (Ika Werk RW-20, Janke and Kunkel), equipped with an impeller of 4 cm in diameter, was positioned 2 cm above the bottom of the beaker and set to 2000 rpm. Next, approximately 0.1 g of SU-8 50 was dropped from a spatula between the impeller and the side of the beaker. If magnetic particles were embedded in the rods,^54^ this was done by thoroughly mixing ∼0.05 g of iron(II, III) oxide nanopowder with ∼0.3 g SU-8 50 before adding it to the mixing vessel. The shear flow resulted in droplet emulsification and stretching, which led to rod formation. For a more detailed account on the rod-formation mechanism, we refer to ref (55). Mixing was continued for 10 min, after which the beaker was covered with aluminum foil to shield it from light and placed in a sonication bath (M2800H-E, Branson) for 1–1.5 h to break the rods into shorter pieces. To make straight rods, 20 mL of the sonicated glycerol solution was placed under UV light (XL-1500 V cross-linker; (6×) 15 W 365 nm, Spectrolinker) for 15 min to cross-link the polymer rods. To make curved rods, 20 mL of glycerol solution was transferred to a large Petri dish and placed approximately 1 m beneath a tube light for 15 min. Then, the solution was placed in a 95 °C oven for 30–40 min, depending on the desired amount of curvature. Longer heating resulted in more highly curved rods. Finally, this solution was exposed to 15 min of UV light to cross-link the polymer rods.

To replace the glycerol with water, rods were repeatedly centrifuged and redispersed in Milli-Q water containing 0.5 wt % Pluronic F108. Centrifugation was performed at 3000*g* for 30 min. Redispersion of particles was achieved through vortex mixing and sonication for 5–10 min. In total, three cycles were performed. To remove large or irregularly shaped rods, the sample was left to sediment for 1 h, after which the precipitate was discarded. This was followed by four cycles of centrifugation at 600*g* for 10 min, where the supernatant was transferred to a separate bottle and the precipitate redispersed for the next cycle. After four cycles, the precipitate was discarded, and the accumulated supernatant was centrifuged at 3000*g* for 30 min. The precipitate of this final centrifugation was redispersed in 5 mL of Milli-Q water containing 0.5 wt % Pluronic F-108 to obtain the desired concentration of rods.

### Optical Imaging and Sample Preparation

The measurements were carried out using an inverted fluorescence microscope (Nikon Eclipse TE2000-U) equipped with a Basler acA4112–30um CMOS camera and an oil objective lens (60×, 1.4 NA). Typically, 20 μL of vesicle solution was added to one of the wells of an 8-well chamber slide (μ-Slide 8 Well Glass Bottom, Ibidi) and left for 10 min to allow the vesicles to settle. In a separate tube, a rod solution was prepared with straight or curved rods, 100 mM glucose, and PAM at double the desired final concentration. Then, 20 μL of this rod-depletant medium was gently added to the vesicle solution in the well using a pipet. Measurements of the vesicle-rod interactions were typically taken at 1–5 fps for several minutes. Vesicle membranes contained a small fraction of fluorescently labeled lipids, while the rods were not fluorescent. Therefore, image acquisition was performed in composite mode (fluorescence + bright field). We estimated the membrane tension of the vesicles using shape fluctuation analysis through flickering spectroscopy, as described in previous studies.^6,7,56^

An attractive interaction between vesicles and the bottom of the well arose from the nonspecific depletion interactions induced by PAM. At high PAM concentrations (≥0.5 wt %), this interaction caused vesicle bursting in uncoated wells. To prevent this, a simple PAM coating was applied to the wells (SI, Section S5). This coating did not fully prevent vesicle adhesion but did prevent bursting. To apply the coating, a droplet of 1 wt % PAM solution was placed on the bottom of the microscope well and left to dry out overnight. Then, to remove excess PAM, the well was washed with Milli-Q water and dried with an N_2_ airflow before using for experiments.

## Supporting Information Available

Additional experimental details, supporting data, and theoretical analysis (PDF)
Movie S1: Partially wrapped rods in the tip-wrapped and the side-wrapped state (MP4)
Movie S2: Partially wrapped rods with round tips spontaneously transitioning from the tip- to the side-wrapped state (MP4)
Movie S3: Magnetically responsive rod transitioning from the tip- to the side-wrapped state (and vice versa) (MP4)
Movie S4: Rod engulfment along the tip-first and the side-first pathway (MP4)
Movie S5: Step-wise cooperative wrapping of a two-rod cluster (MP4)
Movie S6: Engulfment of a curved and a straight rod by low-tension vesicles (MP4)
Movie S7: Unwrapping of a rod as a result of a hypotonic shock (MP4)
