# Supplementary material for: Role of Shape in Particle-Lipid Membrane Interactions: From Surfing to Full Engulfment
Source: ACS Nano. 2024 Mar 21;18(15):10407–16. doi: 10.1021/acsnano.3c11106 (PMC11025115; doi:10.1021/acsnano.3c11106)
Supplement: Supplementary file 1 — nn3c11106_si_001.pdf [file nn3c11106_si_001.pdf]

Role of Shape in Particle-Lipid Membrane Interactions: From Surfing to Full Engulfment

Supporting Information

Stijn van der Ham,[1] Jaime Agudo-Canalejo,[2,3] and Hanumantha Rao Vutukuri[1,*]

[1] *Active Soft Matter and Bio-inspired Materials Lab, Faculty of Science and Technology, MESA+ Institute, University of Twente, 7500 AE Enschede, The Netherlands*

[2] *Department of Living Matter Physics, Max Planck Insitute for Dynamics and Self-Organization, Göttingen, D-37077, Germany*

[3] *Department of Physics and Astronomy, University College London, London WC1E 6BT, United Kingdom*

EXPERIMENTAL SECTION

S1. Characterization of SU-8 microrods

To characterize particle morphology, we employed scanning electron microscopy (SEM). For SEM sample preparation, a solution of rods in water was dried on a silicon wafer and subsequently coated with 5 nm of platinum/palladium. Imaging was performed using a JEOL JSM-6010LA scanning electron microscope at 5 kV. We analyzed the length, diameter, and tip shape of the rods, and assessed any deformation caused by the embedding of magnetic particles. Rod lengths and diameters were measured manually using ImageJ FIJI, based on images taken at x500 and x2000 magnifications, respectively.

The average lengths and diameters of straight and curved rods were measured, with the lengths based on 166 straight and 258 curved rods, and the diameters based on 197 straight and 146 curved rods. The average lengths were 4.4 ± 2.5 μm for straight rods and 4.1 ± 2.7 μm for curved rods, while the average diameters were 0.39 ± 0.09 μm and 0.40 ± 0.09 μm for straight and curved rods, respectively. The error represents the standard deviation in each case. Figure S1a and S1b present the corresponding probability distributions for the lengths and diameters, respectively.

The tip shapes of the rods were extracted from the SEM images. The majority of straight rods had flat tips, resembling the end of a cylinder, as shown in the inset of Fig. S1c, while the majority of curved rods had hemispherical tips, as shown in the inset of Fig. S1d. Notably, some rods fabricated using the curved method exhibited minimal curvature along their length but retained hemispherical end-caps.

Magnetically responsive rods were fabricated by embedding Fe(II,III)O nanoparticles inside the rods during their production.[1] Under an optical microscope, these embedded particles are visible as black dots within the rod, as depicted in Fig. S1f. To assess the impact of embedding these nanoparticles on the shape of the rods, specifically regarding bulging, we compared the SEM images of rods with and without magnetic particles. As shown in Fig. S1e, the inclusion of magnetic particles caused minor bulging in the rods, which was not present in the rods without nanoparticles. Given the minimal extent of the bulging, we inferred that it does not significantly affect the rods' engulfment.

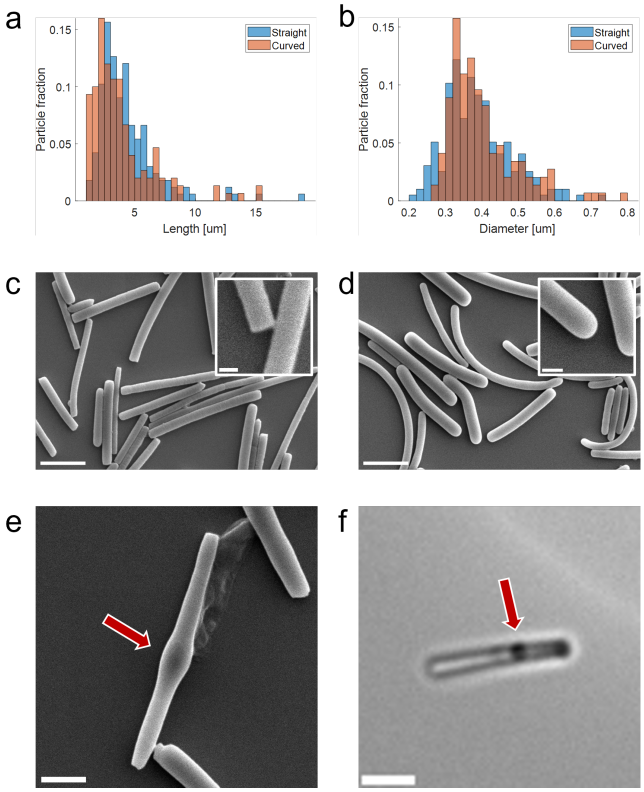

FIG. S1. Characterization of the rod-like particles. a,b) Length (a) and diameter (b) distribution of rods as determined from SEM images. c) SEM image at 2000x magnification of straight rods, inset shows the flat tip. d) SEM image at 2000x magnification of curved rods, inset shows the round tip. e,f) SEM (e) and composite (bright-field + fluorescence) microscopy image (f) of a rod with a magnetic particle embedded. scale bars represent 2 μm for c,d and f, and 1 μm for e. The scale bars in the inset in c and d represent 0.2 and 0.4 μm, respectively.

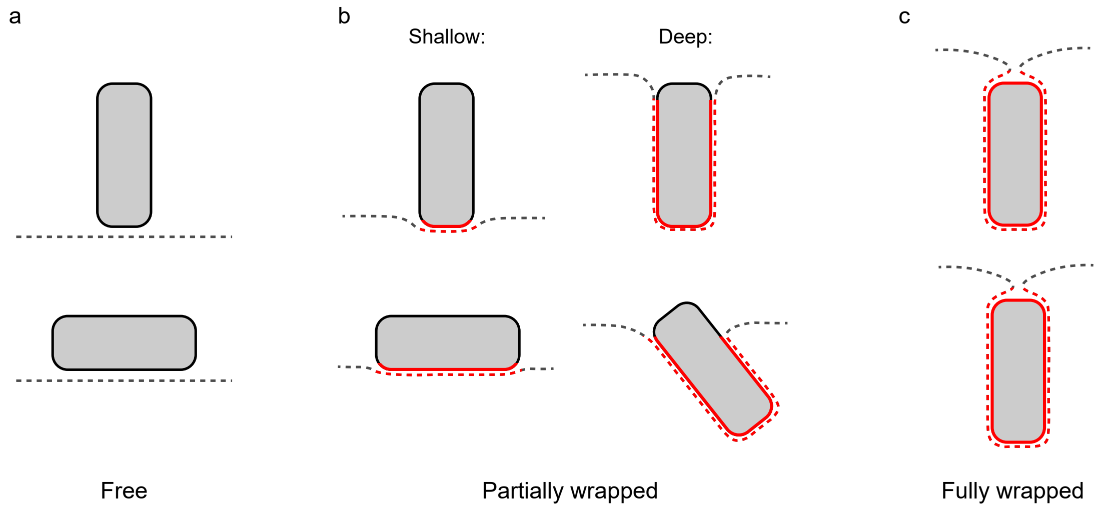

FIG. S2. Schematic representation of the free (a), partially wrapped (b), and the fully wrapped (c) state. The red lines depict the contact region between the rod and the membrane. The top and bottom row correspond to the tip-first and side-first engulfment pathway, respectively.

S2. Depletion interaction: overlap volume in the free, partially, and fully wrapped state

In the partially wrapped state, the rod is partly enveloped by the membrane such that only a fraction of the rod is wrapped, $f = A_{\rm co}/A_{\rm rod}$ with $0 < f < 1$, where $A_{\rm co}$ is the area of contact between the rod's surface and the vesicle membrane, as illustrated respectively by the red solid and dashed segments in Fig. S2. Furthermore, the value of f can vary, leading to what we term shallow-wrapped (for f is close to 0) and deep-wrapped states (for f is close to 1). The free/surfing state corresponds to $f = 0$, and the fully wrapped state corresponds to $f = 1$.

When the particle and the vesicle are not in contact (free state), the excluded volume is given by $V_{\rm free} = V_{\rm ves} + V_{\rm rod} + A_{\rm ves}R_G + A_{\rm rod}R_G$. Here, the third term represents the volume of a layer of thickness R_G around the membrane (black dashed line in Fig. S2a), and the fourth term the volume of a layer of thickness R_G around the particle surface (black solid line in Fig. S2a). In a partially wrapped state, the excluded volume is $V_{\rm pw} = V_{\rm ves} + V_{\rm rod} + (A_{\rm ves} - A_{\rm co})R_G + (A_{\rm rod} - A_{\rm co})R_G$. Here, the third term represents the volume of a layer of thickness R_G around the part of the membrane not bound to the particle (black dashed line in Fig. S2b), and the fourth term represents the volume of a layer of thickness R_G around the part of the particle surface not bound to the membrane (black solid line in Fig. S2b). The reduction in excluded volume is then $V_{\rm ov} = V_{\rm free} - V_{\rm pw} = 2R_GA_{\rm co}$. Note that we can also use the wrapping fraction f to write $V_{\rm ov} = 2R_GA_{\rm rod}f$.

In the fully wrapped state (Fig. S2c), which corresponds to the limit $A_{\rm co} \to A_{\rm rod}$ (or equivalently $f \to 1$), the excluded volume is $V_{\rm fw} = V_{\rm ves} + V_{\rm rod} + (A_{\rm ves} - A_{\rm rod})R_G$, and $V_{\rm ov} = V_{\rm free} - V_{\rm fw} = 2R_GA_{\rm rod}$.

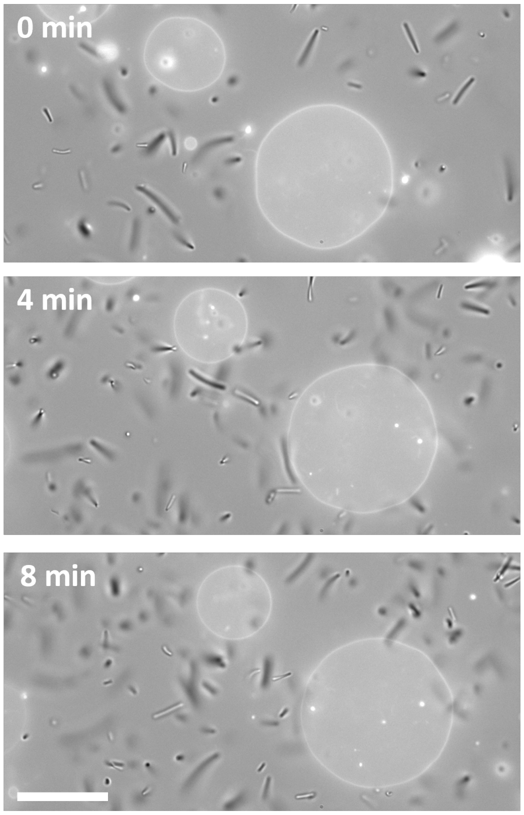

FIG. S3. Time-lapse of composite (bright-field + fluorescence) microscopy images of vesicles and rods in the absence of any added depletant over the course of 8 min. The scale bar represents 10 μm.

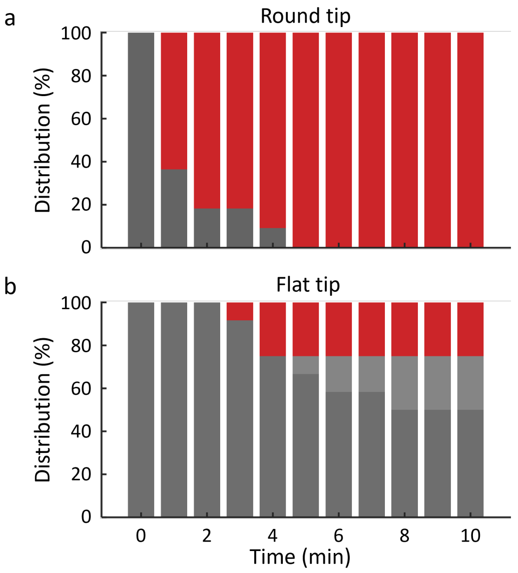

FIG. S4. Fraction of rods that is in the tip- (dark grey) or side-wrapped (red) state, or sedimented (light grey) for round-tipped (a) and flat-tipped (b) rods as a function of time since the removal of the magnet.

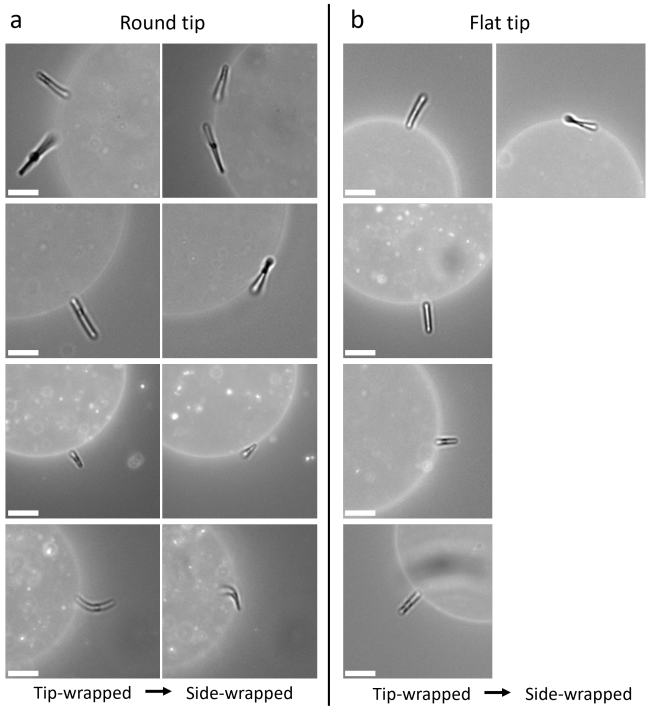

FIG. S5. Composite (bright-field + fluorescence) images of round-tipped (a) and flat-tipped (b) rods before and after their transition from the tip- to the side-wrapped state. For the rods which did not undergo a transition the second image is absent. The scale bar represents 5 μm in all images.

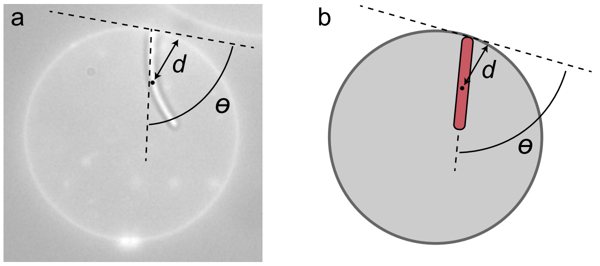

FIG. S6. Measurement of the angle and the distance of the rod relative to the membrane. a) Composite (bright-field + fluorescence) image of a curved rod. Indicated are the angle, θ, and distance, d. b) Schematic depiction of a straight rod with the same angle and distance measurements.

S3. Angle and distance measurement during engulfment

The angle θ was defined as the angle between the long axis of the rod and the tangent of the membrane at the point of contact (Fig. S6). For curved rods, the tangent of the rod at the membrane contact point was considered instead of the long axis (Fig. S6a). Notably, angles between the rod and the membrane were consistently treated as positive values. The distance was defined as the shortest distance between the center of mass of the rod and the membrane. The distance was considered positive if the center of mass was positioned outside the vesicle, while it was recorded as negative if the center of mass was situated inside the vesicle.

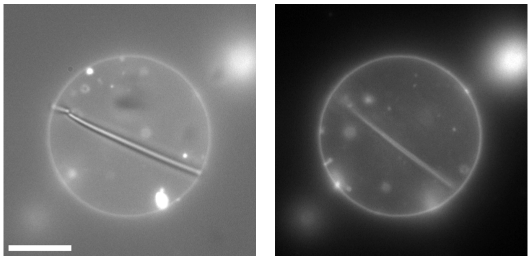

FIG. S7. Composite (bright-field + fluorescence) image (a) and fluorescence image (b) of a high aspect ratio rod in the fully wrapped state. The rod has a length that is approximately equal to the diameter of the vesicle and is fully wrapped. What is more, it is engulfed in a tube together with a much shorter rod. The scale bar represents 10 μm.

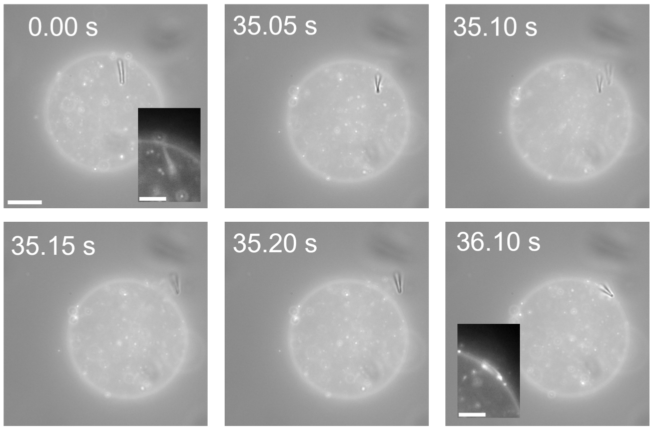

FIG. S8. Time-lapse of composite (bright-field + fluorescence) images of a rod unwrapping due to an increase in membrane tension through an osmotic shock. The insets show the fluorescence image. The scale bar represents 10 μm, and 5 μm for the insets.

S4. Unwrapping

The (partial) unwrapping of fully wrapped rods was achieved by diluting the outside glucose concentration from 100 mM to 83 mM, while keeping the PAM concentration (0.5 wt.-%) constant. The dilution of the outside glucose concentration lead to an osmotic imbalance, which increased the vesicle membrane tension. The increased membrane tension made the fully wrapped state unfavourable, hence causing the rod to unwrap.

Figure S8 shows an example of an unwrapping transition. The unwrapping occurs on the order of tens of milliseconds and causes the rod to be shot out of the vesicle. However, instead of completely detaching from the membrane, the rod is retracted by the vesicle, ending up in a side-wrapped state. This retraction suggests that the membrane is still attached to the rod.

S5. PAM coating

Figure S9a-b shows the comparison between the bottom of an uncoated and coated well after letting rods in 0.5 wt.-% PAM solution sediment for 30 min. In the uncoated well, rods and small clusters of rods are present that adhere strongly to the bottom. Contrastingly, in the coated well, there is a clear reduction in the attractive interaction of rods with the wall, while the attractive interaction between rods remains the same. This is evidenced by sedimented rod clusters which are not stuck to the bottom.

For vesicles, the coating effectively prevents bursting and increases the number of floppy vesicles that are present in the sample. An example of a burst vesicle is shown in Fig. S9c. However, the coating does not completely prevent vesicle adhesion, leading to partial adhesion at higher polymer concentrations.

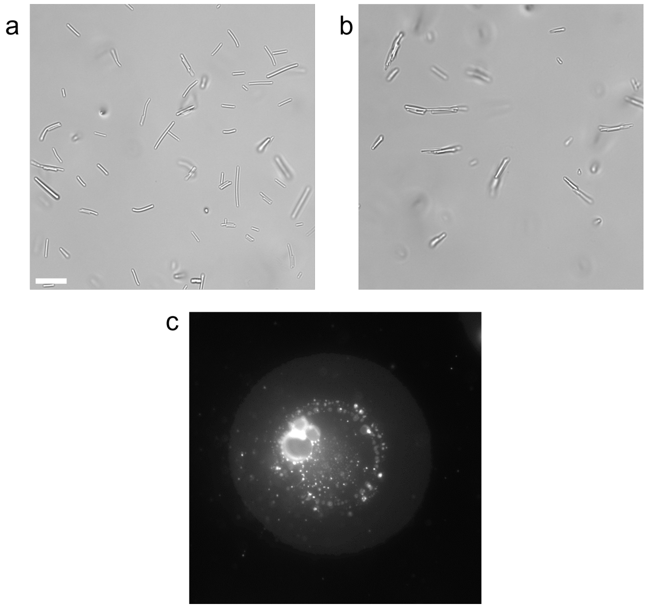

FIG. S9. a,b) Bright-field microscopy images of rods in 0.5 wt.-% PAM solution in an uncoated (a) and coated well (b). The scale bar is 10 μm. c) Burst vesicle in an uncoated well.

S6. Spontaneous curvature

It is worth noting that the simulation and theoretical works often assume that the GUV membranes have zero spontaneous curvature. However, in actual experimental conditions, this is not always the case, as inferred by the observation of inward tubular structures in some of our vesicles. Nevertheless, large spontaneous curvatures (with magnitudes much larger than the inverse particle size) can be approximated as generating an effective "spontaneous tension".[2] Indeed, we find that our experimental results are qualitatively consistent with the theoretical predictions for nonzero tension but zero spontaneous curvature.

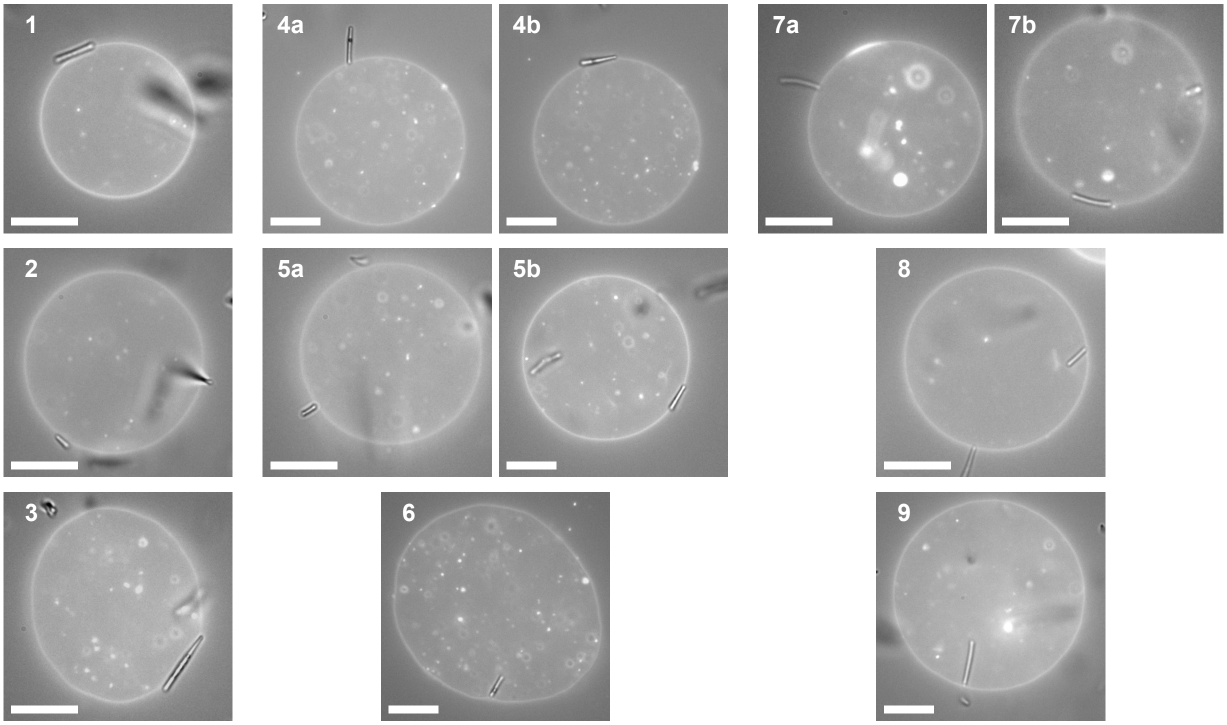

FIG. S10. Composite (bright-field + fluorescence) images of the vesicles in the insets in Fig. 3 of the main text. The scale bars represent 10 μm.

THEORY SECTION

S7. Wrapping of spherocylinders for zero interaction range

We consider a spherocylindrical particle that may have flat tips, as in Fig. S11. A round tip corresponds to $r_{\rm tip} = a$, whereas a flat tip corresponds to $r_{\rm tip} > a$. The area of the particle is given by

$$A = 2\pi a h + 2 \cdot 2\pi r_{\rm tip}^2 \left(1 - \sqrt{1 - (a/r_{\rm tip})^2}\right) \quad \text{(S1)}$$

where the first term gives the area of the cylindrical side, and the second term the area of the tips.

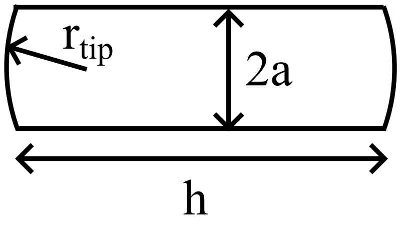

FIG. S11. Spherocylinder geometry. The length of the cylindrical segment is h, and the radius of the cylinder is a. A particle with perfectly round, hemispherical tips would have $r_{\rm tip} = a$. A particle with fully flat tips would have $r_{\rm tip} \to \infty$.

The bending free energy of a spherocylinder shape is given by

$$E_{\rm be} \approx \pi\kappa(h/a) + 8\pi\kappa \quad \text{(S2)}$$

The first term is the bending free energy of the cylindrical side, while the second term is the free energy of a sphere. This second term is exact for the case of hemispherical round tips with $r_{\rm tip} = a$, and serves as an approximation (more precisely, a lower bound) for the bending free energy of the tips in the more general case. A more exact calculation would require us to know how the particle is curved along the rim connecting the tips to the side of the particle (or how the membrane is curved around this rim during engulfment if it does not precisely follow the particle shape at the rim because it is too sharp).

We now assume that the adhesion between the particle and the membrane occurs via a potential with an infinitesimally small interaction range (contact potential). The total free energy of a fully wrapped particle, including bending, tension (with membrane tension Σ), and adhesion (with adhesion strength W) is then

$$E_{\rm W} = E_{\rm be} + \Sigma A - WA \quad \text{(S3)}$$

whereas the free energy of a free particle is $E_{\rm F} = 0$. The fully wrapped state is energetically favored over the free state when $E_{\rm W} < E_{\rm F}$, which implies

$$W > E_{\rm be}/A + \Sigma \equiv W(D) \quad \text{(S4)}$$

which defines the discontinuous transition D. Because the free and shallow partially-wrapped states have similar free energy, this condition also serves as an approximation to the discontinuous transition between partially-wrapped and fully-wrapped state.[3]

Eq. (S4) is just an energetic condition but does not tell us about the dynamic stability of the free state. The stability conditions of the free state have been studied in Ref. 4 for spherical particles and Ref. 5 for nonspherical particles. Assuming the membrane is initially flat before making contact with the particle, a point at the surface of the particle that has mean curvature $M_{\rm pa}$ will become partially wrapped when it touches the membrane whenever

$$W > 2\kappa M_{\rm pa}^2. \quad \text{(S5)}$$

We can, therefore, write two stability conditions that are relevant for the spherocylindrical particle. The side of the particle will become wrapped when it touches the membrane, leading to a partially-wrapped state where the particle is in the side configuration when

$$W > 2\kappa M_{\rm side}^2 = 2\kappa/(2a)^2 = \kappa/(2a^2) \equiv W(L_{\rm side}) \quad \text{(S6)}$$

which defines the stability line $L_{\rm side}$. The tip of the particle will become wrapped when it touches the membrane, leading to a partially-wrapped state where the particle is in the tip configuration, when

$$W > 2\kappa M_{\rm tip}^2 = 2\kappa/r_{\rm tip}^2 \equiv W(L_{\rm tip}) \quad \text{(S7)}$$

which defines the stability line $L_{\rm tip}$.

The lines defined by (S4) and (S6) are used to construct the phase diagram in Fig. 3 of the main text, using $a = r_{\rm tip} = 0.2$ μm, $h = 5$ μm, and $\kappa = 20$ k_BT.

The theory above predicts a difference between particles with a round tip ($r_{\rm tip} = a$) and particles with a sufficiently flat tip ($r_{\rm tip} > 2a$). For particles with a round tip, we find $W(L_{\rm side}) < W(L_{\rm tip})$, which implies that we expect them to first become attached at the sides as the adhesive strength is increased. On the other hand, for particles with a flat tip we find $W(L_{\rm tip}) < W(L_{\rm side})$, implying that we expect them to first attach at the tip, and only later at the side, as the adhesive strength is further increased.

In contrast to this prediction, experiments show that, even for particles with flat tips, side adhesion occurs first with increasing PAM concentration and moreover that the side-adhered state is more stable than the tip-adhered state (as spontaneous transitions from tip-adhered to side-adhered states are observed, but not vice versa). Below, we show that this effect is due to the finite range of the depletion interactions (overlap volume).

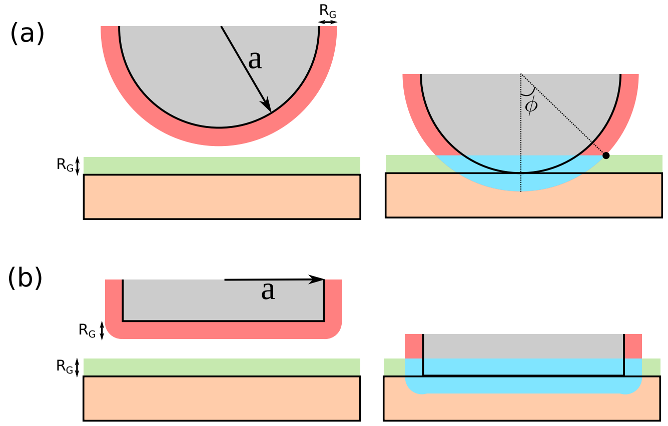

FIG. S12. Geometries for the calculation of overlap volumes in depletion interactions. The radius of the depletant particles is R_G. The particle is drawn in grey (with excluded volume in red) and the flat membrane in orange (with excluded volume in green). The maximum excluded volume overlap (which occurs at contact) is shown in blue. The geometry in (a) is relevant to both the tip-adhesion of rounded-tip rods, in which case the relevant overlap volume is the volume of the blue region when taken to represent an axisymmetric shape with a vertical axis of rotation; and the side-adhesion of rods, in which case the relevant overlap volume corresponds to the area of the blue region times the length of the rod. The geometry in (b) is relevant to the tip-adhesion of flat-tip rods.

S8. Overlap volumes for depletion interactions

The unexpected dominance of side-adhered configurations against tip-adhered configurations at low adhesion can be explained by taking into account the finite range of the depletion interactions that mediate adhesion in the system. Qualitatively, it is sufficient to consider the maximum overlap between the excluded volume regions of the particle and the membrane, which happens when the two come into contact, see Fig. S12.

Let us first consider the situation in Fig. S12a, which is relevant for the the tip-adhesion of rounded-tip rods, and for the side-adhesion of cylindrical rods. At contact, the excluded volume overlap is defined by the angle ϕ which can be calculated from trigonometry as

$$\cos\phi = \frac{a - R_G}{a + R_G}. \tag{S8}$$

For the tip adhesion of rounded-tip rods, we should interpret a as the radius of the hemispherical tip, and the overlap region (in blue) as the volume of the corresponding axisymmetric shape around the vertical axis, which is a spherical cap segment with radius $a+R_G$ and aperture angle ϕ. The volume of this spherical cap segment can be calculated as

$$V_{\text{round-tip}} = \frac{\pi}{3}(a+R_G)^3(2+\cos\phi)(1-\cos\phi)^2 \tag{S9}$$

$$= 4\pi a R_G^2\left(1+\frac{R_G}{3a}\right) \tag{S10}$$

$$= 4\pi a R_G^2 + O(R_G^3). \tag{S11}$$

For the side adhesion of cylindrical rods with length h, we should interpret a as the radius of the rod whose axis is parallel to the membrane (perpendicular to the page), and the overlap region (in blue) as the area of the cross-section of the corresponding volume, which is a segment of a cylinder with radius $a + R_G$ and aperture angle ϕ. The volume of this cylindrical segment is

$$V_{\text{side}} = h\frac{(a+R_g)^2}{2}\left[2\phi - \sin(2\phi)\right] \tag{S12}$$

$$= \frac{16}{3} h a^{\frac{1}{2}} R_G^{\frac{3}{2}} + O\left(\frac{hR_G^{\frac{5}{2}}}{a^{\frac{1}{2}}}\right). \tag{S13}$$

Finally, we consider the tip-adhesion of a flat-tip rod, as in Fig. S12b. The depletion volume in this case is simply

$$V_{\text{flat-tip}} = 2\pi a^2 R_G + O(aR_G^2) \tag{S14}$$

where the dominant term represents the depletion volume of a disk of radius a, and the higher order terms represent the overlap volume around the sharp edges of the tip.

To understand why the side-adhered configuration is more stable than the tip-adhered configuration for flat-tip rods, let us compare the overlap volumes V_{side} and $V_{\text{flat-tip}}$. We find that the side configuration has a larger overlap volume $V_{\text{side}} > V_{\text{flat-tip}}$, and is therefore more strongly adhered, whenever

$$\frac{h}{a} > \frac{3\pi}{8}\sqrt{\frac{a}{R_G}}, \tag{S15}$$

which implies that the aspect ratio of the rod has to be above a critical value. Using $a = 200$ nm and $R_G = 50$ nm, we find this critical aspect ratio to be $\simeq 2.36$. Because our rod particles have a much larger aspect ratio (with $h \sim 5$ μm, giving $h/a \sim 25 \gg 2.36$), the side-adhered configuration is significantly more stable than the tip-adhered configuration in our experiments. Indeed, we find $V_{\text{flat-tip}}/V_{\text{side}} = \frac{3\pi}{8}\frac{a^{\frac{3}{2}}}{hR_G^{\frac{1}{2}}} \approx 0.09$.

The binding energies of the side-adhered state and the tip-adhered state for flat tip rods may be estimated as $\rho k_B T V_{\text{side}}$ and $\rho k_B T V_{\text{flat-tip}}$, respectively, where ρ is the PAM concentration. Because the transition state between the two corresponds to a diagonally-oriented rod which has negligible overlap volume due to the kink-shaped edge of the tip, and therefore negligible binding free energy, the two binding energies respectively correspond to the height of the free energy barrier for the

transition from the side-adhered to the tip-adhered state and vice versa.

For rounded-tip rods, the side-adhered state is much more stable than the tip-adhered state, as we find $V_{\text{round-tip}}/V_{\text{side}} = \frac{3\pi}{4}\sqrt{aR_G}/h \ll 1$. Indeed, for the geometric parameters quoted above, we find $V_{\text{round-tip}}/V_{\text{side}} \approx 0.05$.

Lastly, we note that tip-adhered states are significantly more stable for flat-tip rods than for rounded-tip rods. For the geometric parameters quoted above, we find $V_{\text{round-tip}}/V_{\text{flat-tip}} = 2R_G/a = 0.5$.

SUPPORTING MOVIES

Movie S1: Partial wrapping
Time-lapse movie combining fluorescence and bright-field microscopy to illustrate the two orientations of the partially wrapped state. The left panel displays a flat-tipped rod in the tip-wrapped state, while the right panel displays a flat-tipped rod in the side-wrapped state. Both panels were recorded at a rate of 4 frames per second (fps) and are played back at 10 fps.

Movie S2: Spontaneous rotation
Time-lapse movie combining fluorescence and bright-field microscopy to illustrate the spontaneous transitions of a round-tipped rod from the tip-wrapped state to the side-wrapped state. The left panel displays a curved rod. This segment was recorded at 5 fps and is played back at 20 fps. The right panel displays a straight rod. This segment was recorded at 4 fps and is played back at 16 fps.

Movie S3: Magnetic reorientation
Time-lapse movie combining fluorescence and bright-field microscopy to illustrate how an external magnetic field can manipulate the orientation of a partially wrapped flat-tipped rod, causing transitions between the tip-wrapped and side-wrapped states. The magnet was intermittently removed to demonstrate the stability of the rod in its new orientation after each transition. The movie was recorded at a rate of 5 fps and is played back at 20 fps.

Movie S4: Wrapping pathways
Time-lapse movie combining fluorescence and bright-field microscopy to illustrate the two engulfment pathways. During the movie, the bright-field illumination is momentarily switched off to clearly highlight the membrane's morphology in the fully wrapped state. In the left panel, a flat-tipped rod enters tip-first and remains perpendicular to the membrane. This segment was recorded at 4 fps and is played back at 10 fps. In the right panel, a round-tipped rod enters side-first and rotates from a parallel to a perpendicular position relative to the membrane. This segment was recorded at 1 fps and is played back at 8 fps.

Movie S5: Cooperative wrapping
Time-lapse movie combining fluorescence and bright-field microscopy to illustrate the step-wise cooperative wrapping of a two-rod cluster, with both rods having round tips. The first rod undergoes wrapping from the tip, while the second initially adheres in a side-wrapped state, eventually transitioning to a fully wrapped state. Both rods are ultimately enclosed in the same membrane tube. The movie was recorded at 5 fps and is played back at 50 fps.

Movie S6: Wrapping pathway at low tension
Time-lapse movie combining fluorescence and bright-field microscopy to illustrate the engulfment pathway of a curved rod with round tips (left panel) and a straight rod with flat tips (right panel) by a low-tension vesicle. Both rods enter tip-first and transition directly from a free state to a fully wrapped state. Rods were initially positioned using an external magnet, which was removed upon contact. Both panels were recorded at 5 fps and are played back at 10 fps.

Movie S7: Unwrapping
Time-lapse movie combining fluorescence and bright-field microscopy to illustrate the unwrapping of a rod with round tips as a result of an increase in membrane tension. The rod is ejected from the fully wrapped state, transitioning to the side-wrapped state. The movie was recorded at 20 fps and is played back at 20 fps (left panel) and 5 fps (right panel).

REFERENCES

1. Alargova, R. G.; Paunov, V. N.; Velev, O. D. Formation of polymer microrods in shear flow by emulsification- Solvent attrition mechanism. *Langmuir* **2006**, *22*, 765–774.

2. Agudo-Canalejo, J. Particle engulfment by strongly asymmetric membranes with area reservoirs. *Soft Matter* **2021**, *17*, 298–307.

3. Deserno, M. Elastic deformation of a fluid membrane upon colloid binding. *Physical Review E* **2004**, *69*, 031903.

4. Agudo-Canalejo, J.; Lipowsky, R. Critical particle sizes for the engulfment of nanoparticles by membranes and vesicles with bilayer asymmetry. *ACS nano* **2015**, *9*, 3704–3720.

5. Agudo-Canalejo, J. Engulfment of ellipsoidal nanoparticles by membranes: full description of ori-

entational changes. *Journal of Physics: Condensed Matter* **2020**, *32*, 294001.

* h.r.vutukuri@utwente.nl
